# Prevalence of stroke cognition and health literacy in high-risk populations in Chengdu: a community-based cross-sectional study

**DOI:** 10.3389/fneur.2025.1559851

**Published:** 2025-08-07

**Authors:** Yue Wang, Chun-yu He, Wen-bo Chen, Xiao-qing Jiang, Yan Xie, Yuan-mei Zhao, Ling Yuan

**Affiliations:** ^1^Department School of Nursing, Institute Chengdu Medical College, Chengdu, Sichuan, China; ^2^The Department of Infectious Diseases, First Affiliated Hospital of Chengdu Medical College, Chengdu, Sichuan, China

**Keywords:** stroke, knowledge, health literacy, influencing factors, high risk

## Abstract

**Objective:**

The purpose of this study is to evaluate the awareness of stroke among high-risk populations in Chengdu and explore the factors contributing to it.

**Methods:**

A cross-sectional study was conducted involving 360 individuals identified as being at high risk for stroke, recruited from four community health service centers in Chengdu. Participants were first screened for stroke risk using a technical plan, followed by an assessment of socio-demographic factors and health education status through a pre-designed structured interview questionnaire. The Health Literacy Management Scale (HeLMS) was utilized to evaluate health literacy levels, while a stroke prevention and treatment knowledge questionnaire was used to assess stroke knowledge. Multiple linear stepwise regression analysis was employed to examine the relationship between stroke prevention knowledge and several independent variables.

**Results:**

The mean stroke prevention and treatment knowledge score were 21.95 ± 8.53, with the lowest score in the dimension of stroke management. The results of the regression analysis indicated that the dimensions of information acquisition ability (*β* = 0.330, *p* < 0.001), education level (*β* = 2.233, *p* < 0.001), communication interaction ability (*β* = 0.280, *p* < 0.001), Stroke health education experience (*β* = −0.117, *p* = 0.005), and hypertension diagnosis (β = −0.112, *p* = 0.007) can predict stroke prevention and treatment knowledge.

**Conclusion:**

This study investigated the knowledge level of stroke prevention and treatment and related influencing factors in high-risk population of stroke in Chengdu. Despite the general awareness of the importance of stroke prevention among high-risk individuals in Chengdu, there is a significant deficiency in their ability to identify stroke early and manage it correctly. There is an urgent need for more targeted and accessible health education initiatives.

## 1 Introduction

Stroke is the second leading cause of death and the third leading cause of disability in the world, with 93.8 million stroke survivors and 11.9 million new strokes worldwide. Without urgent action, the number of global deaths due to stroke is expected to increase by 50% to 9.7 million per year by 2050, and the number of stroke survivors is expected to increase by 50% to 9.7 million per year (1). And it is also the primary cause of mortality and disability among adults in China (2). Primary prevention plays a crucial role in reducing the incidence of stroke, and ample knowledge about stroke prevention and treatment is instrumental in this process. Adequate understanding of stroke among individuals is beneficial for the timely recognition of stroke symptoms and seeking medical attention promptly. This can significantly reduce the adverse outcomes associated with delayed medical intervention. Sheng’s research indicates that patients with inadequate knowledge of stroke and low health literacy often face delays in medical decision-making, which can result in missing the optimal window for thrombolysis (3). This underscores the importance of improving health literacy and stroke awareness to ensure that patients can make informed decisions and receive timely treatment.

A review indicates that the public’s understanding of stroke prevention and management is uneven, with a significant range in the reported levels of knowledge. Specifically, 4.4 to 79% of respondents demonstrate a good understanding of stroke in general, 23.6 to 87% are aware of stroke signs and symptoms, and 10.5 to 86.6% recognize stroke risk factors. However, the proportion of individuals who are proactive in taking preventive measures is relatively low, ranging from 2.4 to 72% (4). National surveys in China reveal that while the correct identification rate of stroke is relatively high at 87.1%, the handling rate is 60.9%, indicating room for improvement. There are also significant regional and demographic variations in these rates, underscoring the need for targeted educational interventions (5). The increasing focus on stroke education in China is evident, with some regions establishing expert consensuses to guide public health campaigns on stroke prevention (6). Scholars have already conducted surveys on the level of stroke knowledge among the general population and stroke patients, but there is a scarcity of research concerning high-risk stroke populations in China.

Health literacy is a critical factor in how individuals obtain, understand, and utilize health care information and services, playing a significant role in promoting health and managing diseases (7). Different dimensions of health literacy play distinct roles in individuals’ adoption of healthy behaviors. Research indicates that the stronger an individual’s ability to acquire and comprehend health information, the better their physical health status. At the same time, the stronger their ability to apply health information, the healthier their lifestyle (8). Furthermore, higher health literacy is associated with more extensive health knowledge (9, 10). Over the past few decades, the health literacy levels of Chinese residents have improved, but they remain generally low, with significant disparities across various aspects such as age, education, and occupation (11). This suggests that while progress has been made, there is still a considerable need to address the inequalities and raise overall health literacy levels to ensure better health outcomes for all segments of the population. Understanding the health literacy status of patients at high risk of stroke may help to formulate targeted health education strategies.

The purpose of this study is to investigate the knowledge of stroke and health literacy levels among high-risk stroke populations, understand the related factors, and provide a theoretical basis for the development of targeted primary prevention health education strategies and reduce the incidence of stroke.

## 2 Methods

### 2.1 Study design and participants

This cross-sectional study was conducted in Chengdu, Sichuan Province from October 2021 to March 2022. The respondents were recruited through stratified, multi-stage random sampling. First, two communities were randomly selected, then two community health centers within these areas were randomly chosen. Finally, our survey was conducted using convenience sampling in four community health centers in Wuhou District and Xindu District of Chengdu City. We selected 382 residents from the visiting population of the community health center who were identified as high-risk individuals according to the “Technical Scheme for Screening and Comprehensive Intervention of Stroke Population.” These individuals came for routine examinations or received other health services.

The inclusion criteria were based on the “Technical Plan for Stroke Population Screening and Comprehensive Intervention” (12), which required participants to be aged 40 or above and have at least three of the following eight risk factors: hypertension, dyslipidemia, diabetes, atrial fibrillation or valvular heart disease, a history of smoking, significant overweight or obesity, lack of exercise or light physical labor, and a family history of stroke, without a history of stroke/transient ischemic attack.

The exclusion criteria included individuals with cognitive or psychiatric disorders and those with severe visual, auditory, or speech impairments.

### 2.2 Assessment tool

This study employed a comprehensive questionnaire consisting of four parts to assess various aspects of the high-risk stroke population’s demographics, clinical information, health education status, and knowledge of stroke prevention and treatment.

#### 2.2.1 Demographic and background information questionnaire

The demographic and background information questionnaire encompassed social demographic details such as gender, age, education, marital status, and employment status, as well as clinical information including hypertension and hyperlipidemia.

#### 2.2.2 Health education status survey questionnaire

The health education status survey questionnaire assessed participants’ attitudes toward learning about stroke prevention and treatment, their past experiences with community disease education, their preferred channels for acquiring knowledge, and the content they wished to understand.

#### 2.2.3 Stroke prevention knowledge assessment

Participants’ understanding of stroke knowledge was evaluated using a widely applied stroke prevention knowledge questionnaire among the stroke population in China. Developed by Zhang (13), the questionnaire comprises 8 dimensions and 36 items: daily living, exercise, diet, risk factors, medication, blood pressure monitoring, stroke signs, and stroke management. Each item is scored as “known” (1 point) or “unknown” (0 points), with a total score ranging from 0 to 36, where a higher score indicates a more comprehensive understanding of stroke prevention knowledge. The questionnaire has a Cronbach’s *α* coefficient of 0.812, demonstrating good internal consistency.

#### 2.2.4 Health literacy survey

The Health Literacy Management Scale (HeLMS), originally developed by Jordan (14), was translated and revised by Sun Linhao (15) to form a 24-item scale encompassing 4 dimensions: information acquisition ability, communication interaction ability, desire to improve health, and willingness for economic support. The scale uses a 5-point Likert scale, with each item scored from “strongly disagree” (1 point) to “strongly agree” (5 points). The total score ranges from 24 to 120, with a score above 96 indicating the presence of health literacy, and higher scores indicating better health literacy.

### 2.3 Data collection

Data collection for this study was facilitated through an online survey method. The questionnaire was uploaded to the “Sojump website”^1^ and a QR code was generated for easy access. Eligible participants could scan the QR code and complete the questionnaire on their devices. Participants received consent forms detailing and outlining the study’s procedures, which they acknowledged by selecting “yes” to confirm participation. To prevent duplicate responses, an IP restriction was implemented, allowing each survey to be completed only once from a single IP address. The research team conducted a comprehensive review of the collected questionnaires for quality control, excluding those with response times below 90 s or exhibiting obvious logical inconsistencies.

### 2.4 Ethical consideration

This study has been reviewed and approved by the Ethics Committee of Chengdu Medical College (Protocol Number: 2022NO.08).

### 2.5 Data analysis methods

Data analysis was performed using SAS 9.4 (version 9.4 for Windows, SAS Institute, Inc., Cary, NC, United States) and R (Version 3.4.3). Normality tests were performed for the distribution of scores in each dimension. For normally distributed data, mean and standard deviation (SD) were used, while median, 25th percentile, and 75th percentile were reported for non-normally distributed data. When comparing two groups, the t-test or ANOVA were used for normally distributed continuous variables, while the Wilcoxon-Mann–Whitney test and the Kruskal-Wallis test were applied for non-normally distributed variables. Correlation analysis between dimension scores utilized Spearman correlation coefficients. To determine the independent factors related to the stroke prevention knowledge score, a multiple linear regression analysis was conducted using the stepwise selection method. The dependent variable was the total knowledge score. The independent variables included in the stepwise analysis were: educational level, income level, type of medical insurance, gender, employment status, whether having high blood pressure, whether exercising, whether considering it necessary to learn about stroke prevention knowledge, whether having participated in community stroke health education activities, and the continuous values of each dimension of health literacy. The criteria for variable entry and retention were *p* < 0.05.

## 3 Results

### 3.1 Demographic characteristics

Out of the 382 eligible participants who completed the health literacy and stroke prevention knowledge questionnaires, 22 questionnaires were excluded due to missing data, resulting in an effective response rate of 94.24%. Table 1 presents the sociodemographic characteristics of the 360 participants. The average age was 71.33 ± 8.62 years. Nearly 59.4% had an educational level of primary school or below, 77.8% were married, the vast majority had medical insurance, and 57.8% had a family monthly per capita income of less than 3,000 RMB.

**Table 1:** The characteristics of the high-risk population for stroke (*n* = 360).

Characteristics	Categories	*n*	*N*%
Gender	Male	171	47.5%
	Female	189	52.5%
Age	<60	48	13.3%
	61–70	87	24.2%
	71–80	175	48.6%
	>80	50	13.9%
Education	Elementary education or below	214	59.4%
	Primary secondary education	82	22.8%
	Higher secondary education	35	9.7%
	Higher education	29	8.1%
Spouse	No	80	22.2%
	Yes	280	77.8%
Working status	Working status	12	3.3%
	Not working	348	96.7%
Living status	With spouse and children	114	31.7%
	With spouse	161	44.7%
	With children	77	21.4%
	Live alone	8	2.2%
Monthly income per capita, CNY	<3,000	208	57.8%
	3,000–5,000	106	29.4%
	5,000–7,000	33	9.2%
	>7,000	13	3.6%
Health insurance	UEBMI	162	45.0%
	URBMI	195	54.2%
	Other	3	0.8%
Hypertension	Yes	299	83.1%
	No	61	16.9%
Hyperlipidemia	Yes	212	58.9%
	No	148	41.1%
Hyperglycemia	Yes	151	41.9%
	No	209	58.1%
Smoking	Yes	92	25.6%
	No	268	74.4%
Atrial fibrillation	Yes	72	20.0%
	No	288	80.0%
BMI (kg/m^2^)	≥24	159	44.2%
	18.5–24	193	53.6%
	<18.5	8	2.2%
Lack of exercise	Yes	229	63.6%
	No	131	36.4%
Family history of stroke	Yes	99	27.5%
	No	261	72.5%

UEBMI, Residents Basic Medical Insurance; UEBMI, Urban Employment Basic Medical Insurance.

### 3.2 Attitudes and needs for health education

**Figure 1:**
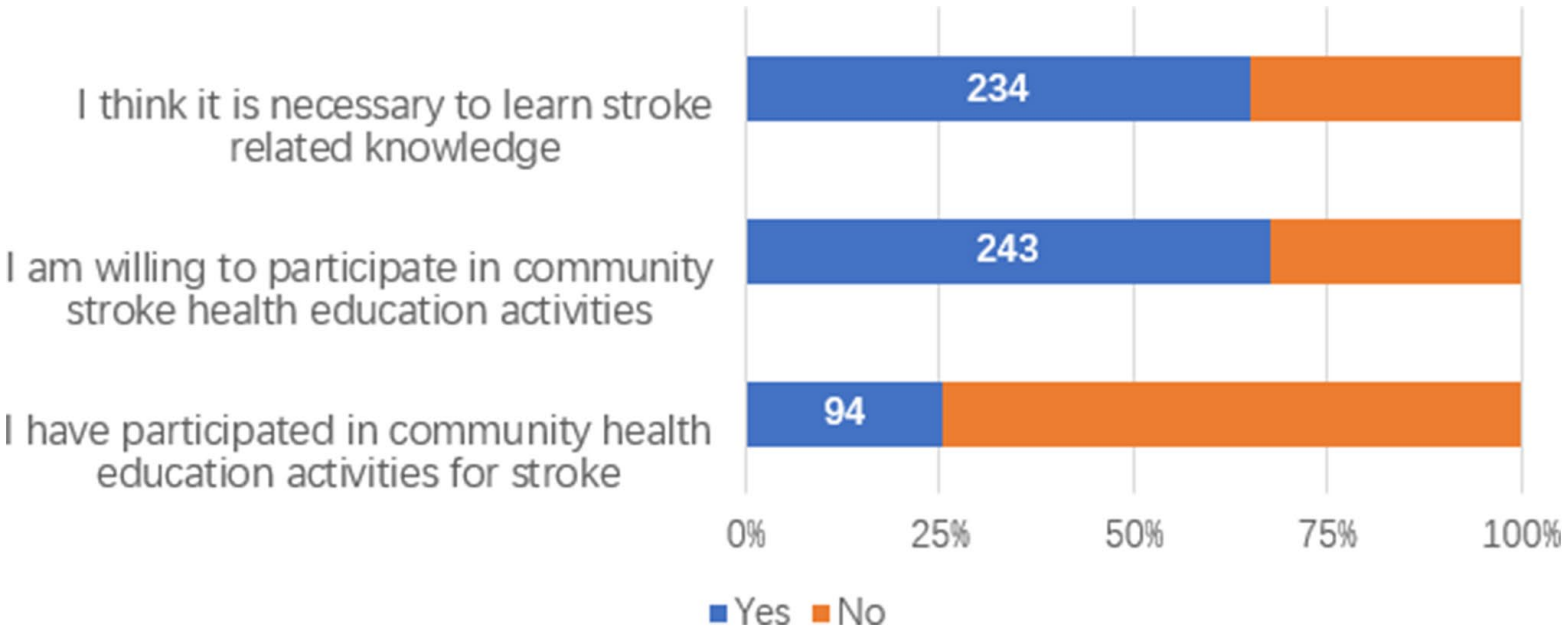
Attitudes toward stroke health education.

**Figure 2:**
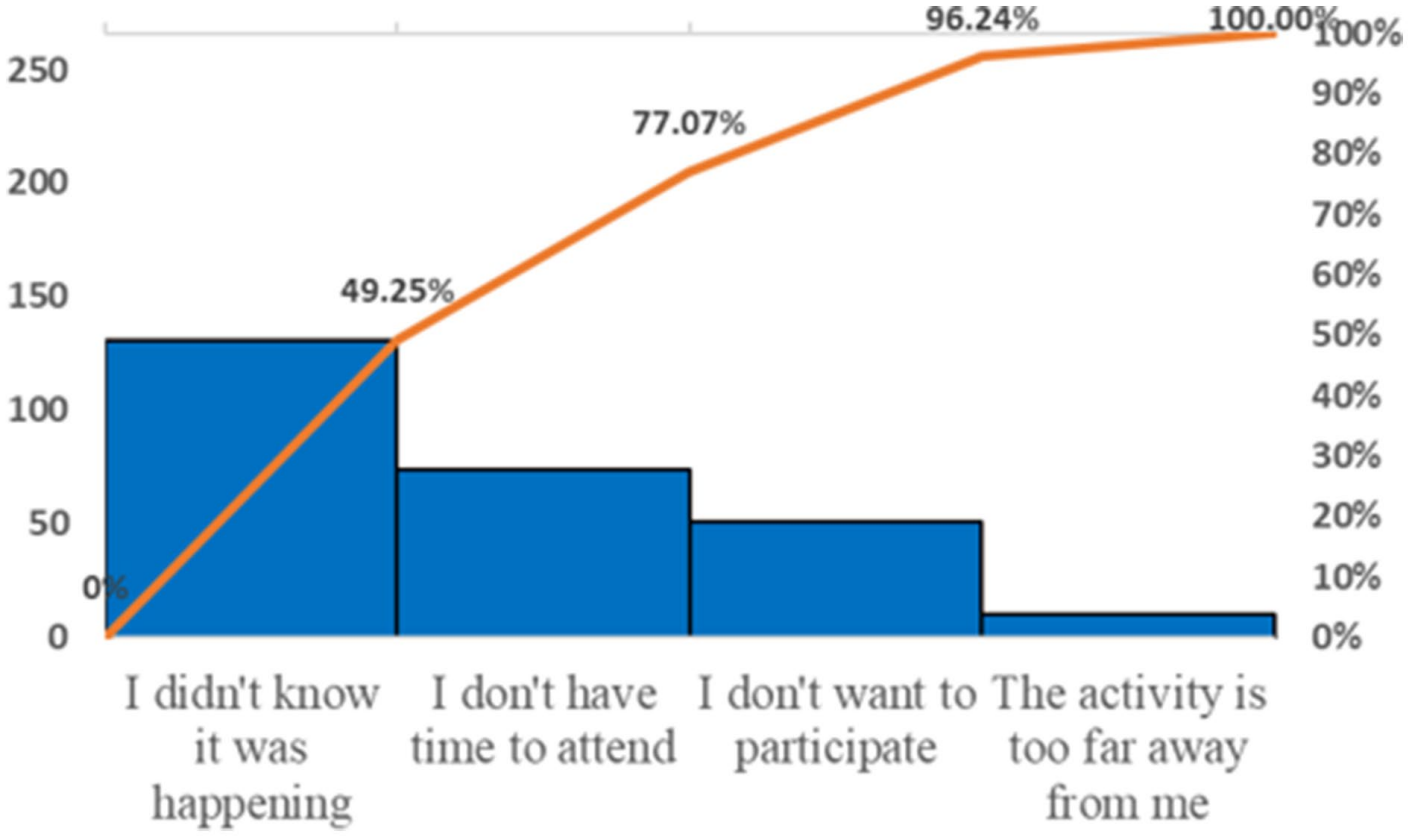
Reasons for respondents not participating in community stroke health education activities.

**Figure 3:**
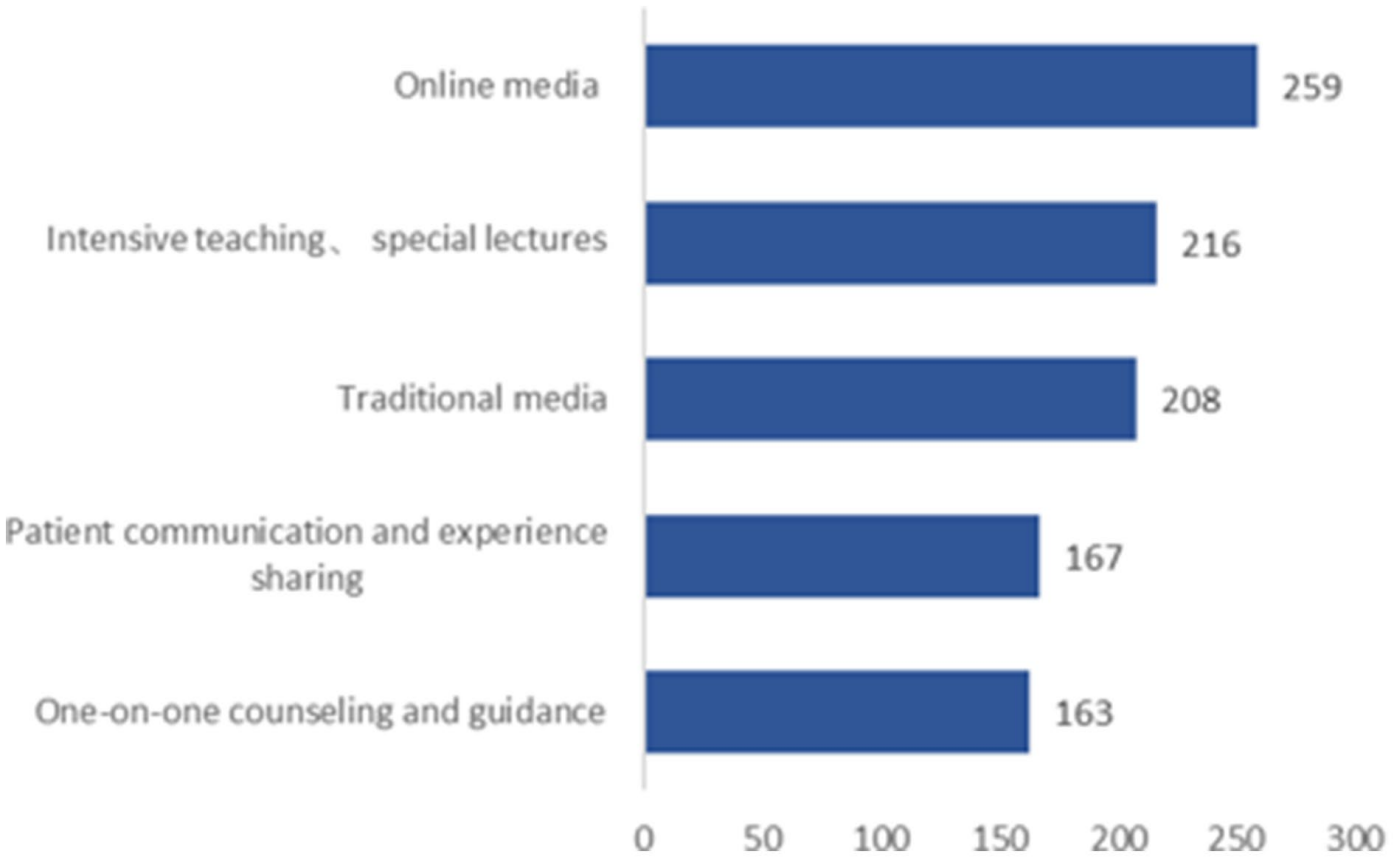
Access to information desired by respondents.

**Figure 4:**
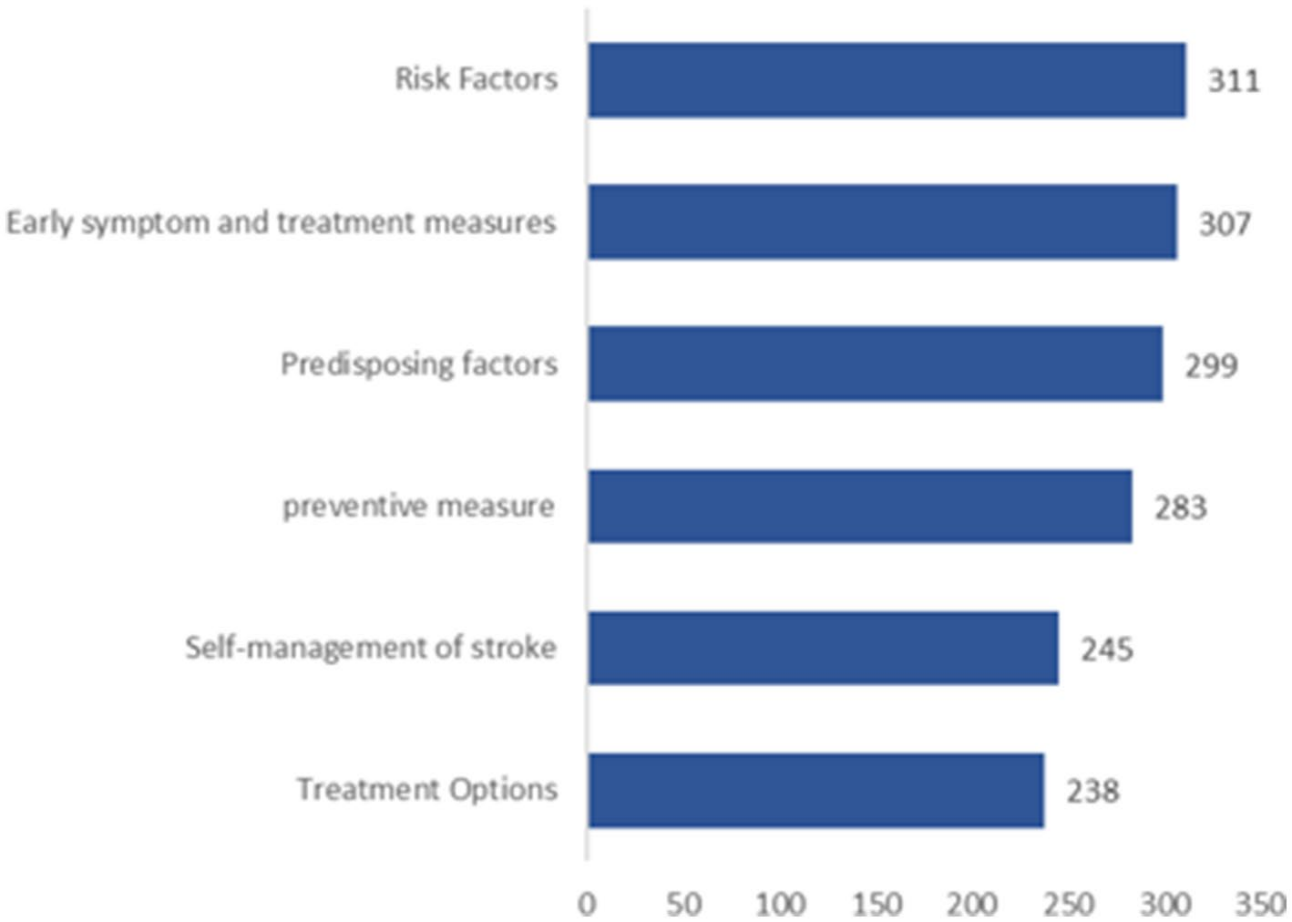
Type of stroke knowledge desired by respondents.

65.00% of the participants believed it was necessary to learn about stroke-related knowledge, and 67.50% were willing to attend community-organized stroke health education lectures. However, only 26.11% had actually participated, with nearly one-third being unaware of such events (Figures 1, 2). “Television, promotional brochures/boards, newspapers, magazines, and professional books” were the most frequently chosen channels for acquiring knowledge, followed by “concentrated teaching and special lectures” (Figure 3). “Risk factors” were the most desired content to learn about, followed by “early symptoms,” “etiologic factors,” “preventive measures,” “post-stroke self-management,” and “treatment plans” (Figure 4).

### 3.3 Health literacy

Only 31.7% of our population had adequate health literacy, with an average score of 90.26 ± 15.46. The highest scoring dimension was the desire to improve health (81.49%), while the lowest was the ability to obtain information (68.54%). Table 2 displays the level of health literacy scores among the participants.

**Table 2:** Health literacy levels of the respondents (*n* = 360).

Dimensions of the scale	Mean	SD	Scoring rate
Health literacy score	90.26	15.46	/
Ability to access information	30.84	7.88	68.54%
Communication and interaction skills	35.07	6.34	77.96%
Improving health intentions	16.3	2.89	81.49%
Willingness for economic support	8.05	1.52	80.50%

Scoring rate was calculated as follows: sum of survey scores of this dimension/(360*total score of this dimension) * 100%.

### 3.4 Stroke prevention and treatment knowledge

#### 3.4.1 Score

The average score for stroke prevention and treatment knowledge was 21.95 ± 8.53, with an overall scoring rate of 60.94%. Among the various dimensions, the highest awareness rate was in the diet dimension (88.96%), and the lowest was in the stroke management dimension (41.39%). Table 3 displays the stroke prevention and treatment knowledge scores of the participants.

**Table 3:** The knowledge scores of stroke prevention and treatment (*n* = 360).

Dimensions of the scale	Mean	SD	Scoring rate^a^
Stroke knowledge score	21.95	8.53	60.94%
Knowledge of daily life	5.43	2.18	68.12%
Knowledge of sports	2.94	1.31	73.54%
Diet knowledge	3.56	0.91	88.96%
Knowledge of Risk factors	2.50	1.81	49.94%
Knowledge of medication	2.79	1.68	55.78%
Blood pressure monitoring knowledge	1.21	0.86	60.42%
Knowledge of stroke warning signs	2.70	2.29	44.95%
Stroke management knowledge	0.83	0.90	41.39%

^a^Scoring rate was calculated as follows: sum of survey scores of this dimension/360 * 100%.

The results of univariate analysis of the influence of sociodemographic and clinical data on the level of stroke knowledge are shown in Table 4. Compared to those with lower education levels, individuals with higher education were more likely to possess adequate stroke prevention and treatment knowledge (*p* < 0.001). Similarly, those with higher incomes were more likely to have more comprehensive knowledge (*p* < 0.001). People with urban employee basic medical insurance were more likely to have more adequate knowledge compared to other types of medical insurance (*p* < 0.001). Male (*p* = 0.005), those who are employed (*p* = 0.007), diagnosed with hypertension (*p* < 0.001), not lacking in exercise (*p* = 0.018), who believe it is necessary to learn about stroke prevention and treatment knowledge (*p* = 0.005), and those who have participated in community stroke health education activities (*p* = 0.005) were found to have more adequate stroke prevention and treatment knowledge.

**Table 4:** Univariate analysis of stroke knowledge scores by demographic and health factors (*n* = 360).

Characteristics		Mean	SD	*p*
Gender				**0.005** ^**^
	Male	23.28	8.44	
	Female	20.75	8.46	
Age				0.074
	<60	24.50	8.54	
	61–70	22.48	8.49	
	71–80	20.97	8.20	
	>80	22.02	9.36	
Education				**<0.001** ^**^
	Elementary education or below	19.13	7.98	
	Primary secondary education	24.39	7.52	
	Higher secondary education	26.66	7.63	
	Higher education	30.24	6.16	
Spouse				0.082
	No	20.49	7.82	
	Yes	22.37	8.69	
Occupation				**0.007** ^**^
	Unemployed or Retired	28.42	7.38	
	Temporary job or Permanent job	21.73	8.49	
Living status				0.06
	With spouse and children	22.47	8.27	
	With spouse	22.57	8.91	
	With children	19.66	8.02	
	Live alone	24.25	6.36	
Monthly income per capita				**<0.001** ^**^
	<3,000	19.71	8.25	
	3,000–5,000	24.18	8.01	
	5,000–7,000	26.55	7.81	
	>7,000	28.08	7.08	
Health insurance				**<0.001** ^**^
	UEBMI	24.46	8.37	
	URBMI	19.99	8.03	
	Other	14.00	14.00	
Hypertension				**<0.001** ^**^
	Yes	22.66	8.50	
	No	18.48	7.88	
Hyperlipidemia				0.841
	Yes	21.88	8.55	
	No	22.06	8.54	
Hyperglycemia				0.369
	Yes	21.48	8.82	
	No	22.30	8.32	
Smoking				0.254
	Yes	21.08	8.51	
	No	22.25	8.54	
Atrial fibrillation				0.597
	Yes	22.42	8.23	
	No	21.84	8.62	
BMI (kg/m^2^)				0.417
	≥24	22.04	7.91	
	18.5–24	22.04	9.04	
	<18.5	18.00	7.67	
Lack of exercise				**0.014**
	Yes	21.15	8.85	
	No	23.36	7.79	
Family history of stroke				0.171
	Yes	22.99	8.98	
	No	21.56	8.34	
I think it is necessary to learn stroke related knowledge				**0.005** ^**^
	Yes	22.87	8.72	
	No	20.25	7.92	
I am willing to participate in community stroke health education activities				0.789
	Yes	22.04	8.49	
	No	21.78	8.65	
I have participated in community health education activities for stroke				**0.005** ^**^
	Yes	24.07	8.37	
	No	21.20	8.48	

Note: ^**^p < 0.05.Abbreviations: URBMI, Residents Basic Medical Insurance; UEBMI, Urban Employment Basic Medical Insurance.

#### 3.4.2 Correlation between stroke prevention knowledge and health literacy sub-domains

Pearson analysis result indicated that stroke prevention and treatment knowledge was positively correlated with information acquisition ability (r = 0.554, *p* < 0.001), communication interaction ability (r = 0.422, *p* < 0.001), desire to improve health (r = 0.346, *p* < 0.001), and willingness for economic support (r = 0.379, *p* < 0.001). Table 5 presents these correlations.

**Table 5:** Pearson analysis of stroke prevention knowledge scores and health literacy sub-domains.

	1	2	3	4	5
1. Stroke prevention knowledge scores	1				
2. Information acquisition ability dimension	0.554^**^	1			
3. Communication and interaction ability	0.422^**^	0.514^**^	1		
4. Improve the health intention dimension	0.346^**^	0.453^**^	0.713^**^	1	
5. Economic support willingness dimension	0.379^**^	0.435^**^	0.604^**^	0.656^**^	1

Note: ^**^p < 0.05.

#### 3.4.3 Influencing factors of stroke prevention and treatment knowledge level

To identify the predictors of stroke prevention and treatment knowledge levels within the population, all significant variables from the bivariate analysis were entered into a stepwise regression model. Stroke prevention and treatment knowledge served as the dependent variable, while education level, income level, type of medical insurance, gender, employment status, hypertension diagnosis, exercise habits, perceived necessity of learning about stroke prevention and treatment knowledge, participation in community stroke health education activities, and various dimensions of health literacy were included as independent variables. The normality probability plot and scatterplots of residuals showed that the data met the basic assumptions of linear regression analysis for normality, linearity, and homoscedasticity. The results of the regression analysis indicated that the dimensions of information acquisition ability (*β* = 0.330, *p* < 0.001), education level (β = 2.233, *p* < 0.001), communication interaction ability (*β* = 0.280, *p* < 0.001), Stroke health education experience (β = −0.117, *p* = 0.005), and hypertension diagnosis (β = −0.112, *p* = 0.007) can predict stroke prevention and treatment knowledge. Information acquisition ability dimension was the largest predictor variable of stroke prevention and treatment knowledge. Detailed results are shown in Table 6.

**Table 6:** The results of multiple linear stepwise regression analysis of stroke prevention and treatment knowledge.

	B	*SE*	*β*	*t*	*P*	95% CI for B	
						Lower	Upper
Constant	0.384	2.212		0.157	0.875		
Information acquisition ability dimension	0.330	0.060	0.305	5.506	<0.001	0.212	0.448
education (Ref: Primary school and below)	2.233	0.428	0.249	5.213	<0.001	1.391	3.076
Communication and interaction ability	0.280	0.066	0.208	4.271	<0.001	0.151	0.409
Stroke health education experience (Ref: Yes)	−2.278	0.804	−0.117	−2.833	0.005	−3.860	−0.697
Hypertension (Ref: Yes)	−2.539	0.943	−0.112	−2.692	0.007	−4.394	−0.684

Model: F = 47.486, *p* < 0.001. VIF: 1.016–1.812; R^2^ = 0.401, adjusted R^2^ = 0.393; SE, standard error; β, standardized coefficient; CI, confidence interval.

## 4 Discussion

This study aimed to assess the level of understanding of stroke prevention and treatment knowledge among high-risk populations in Chengdu communities. The results indicate that the average score for stroke prevention and treatment knowledge among the surveyed population was 21.95 ± 8.53, with an overall scoring rate of 60.94%, which is comparable to findings from other regions (16–18). Participants demonstrated a good understanding of diet and exercise-related knowledge, but the awareness rates for the management and symptom dimensions were lower, suggesting that this population may lack early recognition capabilities for stroke and might not have the correct response measures when facing a stroke. This is similar to the findings of a study conducted in Saudi Arabia (19). Approximately only one-third of the individuals were aware that visual changes and choking while swallowing are also signs of stroke, which aligns with previous research (20, 21). Less than half of the participants realized the importance of immediate bed rest, calling an ambulance, and going to the hospital within 3 h after a stroke occurs. Based on the high incidence rate and poor prognosis of stroke, educational initiatives targeting high-risk populations should urgently intensify publicity regarding stroke symptom recognition and management measures, thus avoiding delays in seeking treatment (22).

Although over half of the participants acknowledged the necessity of learning about stroke-related knowledge and expressed willingness to participate in similar educational activities, less than a third had actually attended, a proportion significantly lower than what would be ideal. A significant number of people did not attend the event because they did not have time, were too far away, or did not know the news. Participants showed a greater interest in learning about “prevention” aspects of stroke. The study also investigated participants’ preferences for learning channels, revealing a preference for online methods such as “WeChat groups/official accounts, short videos, and microblogs” over traditional media like “television, brochures/boards, newspapers, magazines, and professional books.” The preference for “concentrated teaching and special lectures” was also higher than for “one-on-one consultations” and “peer discussions.” This survey provides valuable guidance for subsequent health education activities. Understanding the preferences and barriers of the high-risk stroke population can help tailor educational initiatives to be more effective and accessible. By leveraging preferred channels and addressing the barriers to participation, future programs can better engage the community and enhance stroke prevention and management knowledge.

Our study’s results reveal a significant correlation between health literacy and disease knowledge levels, consistent with similar research (23–25). The dimensions of information acquisition ability and communication interaction ability are crucial predictors of stroke prevention knowledge levels. However, these are also the dimensions with the lowest scores in our study, likely related to the majority of our subjects being elderly with educational levels primarily below primary school, who are often disadvantaged in health information acquisition. The health status and physical functions of the elderly deteriorate with age, and they wish to invest more time and effort to improve their health but are constrained by limited accessible information channels and numerous obstacles in searching for online health information (26). Education level is a significant factor affecting health literacy. Individuals with higher education not only possess better reading, comprehension, and reasoning abilities but are also more inclined to search for health information through online channels and have a stronger ability to discern the credibility of health information (27). Individuals with lower education levels are at a distinct disadvantage in acquiring disease knowledge. On one hand, the explosive growth of online health information requires a certain level of eHealth literacy to quickly and accurately obtain the needed information. On the other hand, while the internet provides a wealth of easily accessible information, the lack of strict content and identity review on platforms leads to a mix of information quality and reliability, necessitating sufficient critical health literacy (6). Research demonstrates the necessity of providing support to stroke high-risk populations to enhance their self-management behaviors (28, 29). For populations with low health literacy, it is essential to not only strengthen early identification and proper handling of stroke knowledge education to enhance individual sensitivity to anomalies but also to train them in information search skills and the ability to discern false information. Recommending reliable health information sources can improve their health information search skills, encouraging proactive health information-seeking behavior to meet their disease management knowledge needs. Additionally, involving family members in the health management of stroke-prone individuals is feasible. Family members can provide emotional support, stimulating the individual’s willingness to express their needs (30, 31). Furthermore, through the family’s substitute online health information search behavior, it may partially compensate for the stroke-prone individual’s information search deficiencies, meeting their information needs (28–34).

A significant finding from this study is that participants who had attended community stroke health education activities demonstrated significantly higher stroke knowledge compared to those who had not. This result aligns with previous research (35–39), highlighting the substantial effectiveness of health education in enhancing the public’s understanding of stroke risk factors, warning signs, preventive measures, and treatment options. However, only 26.11% of our surveyed individuals had participated in stroke education activities, which is lower than the findings in Wahab’s study (40). This suggests that there is considerable room for improvement in the popularization of health education. The rise of digital media is increasingly complementing traditional media in the realm of health information dissemination. Its characteristics of rapid spread and wide reach hold tremendous potential for raising health awareness. As previously mentioned, digital media is the preferred channel for participants to acquire knowledge, which could potentially address the issue of low penetration in traditional health education outreach.

Consistent with a study from Korea (41), our research found that hypertensive patients are associated with higher levels of stroke prevention knowledge. This correlation may be attributed to the heightened perception of adverse outcomes related to hypertension, which motivates these patients to actively seek health information (42). We also analyzed whether other stroke risk factors influence an individual’s level of stroke prevention knowledge. Contrary to expectations, aside from hypertension, the impact of other risk factors on individual stroke prevention knowledge levels was not significant. The reason for this outcome might be that the questionnaire used in this study included multiple items related to hypertension, while other factors such as hyperglycemia and hyperlipidemia were less frequently mentioned. As a result, hypertensive patients had an advantage when responding to the questionnaire.

This study has several limitations. First, the findings are derived solely from an investigation conducted in Chengdu, Sichuan Province, and may not be representative of the situation across the entirety of China. Second, as the included sample predominantly consisted of older adults, and considering the trend of stroke occurring at younger ages, the results may not be generalizable to the younger and middle-aged stroke population. Third, since the sampling method involved selecting participants only from specific healthcare institutions, this restricts the broader applicability of our findings. Systematic differences might exist between individuals who actively utilize community healthcare services and those who do not. These limitations affect the generalizability of the study’s results and indicate the need for multicenter studies involving larger, more diverse populations to validate the findings. Furthermore, a key limitation of this study is that the assessment of participants’ knowledge levels and health literacy relied entirely on self-reporting, which might introduce a degree of motivational bias. Finally, due to its cross-sectional design, this study cannot establish causal relationships between the independent variables and the outcomes.

## 5 Conclusion

In this study, it was observed that although high-risk stroke individuals in Chengdu are generally aware of the importance of stroke prevention, there is a significant deficiency in their ability to early identify and properly manage stroke incidents. This highlights an urgent need for more targeted and accessible health education initiatives. Future interventions should focus on enhancing health literacy, particularly in the areas of information acquisition and communication skills. Leveraging the potential of online media can significantly improve the effectiveness of reaching and educating the public.

## Data Availability

The original contributions presented in the study are included in the article/supplementary material, further inquiries can be directed to the corresponding author.

## Footnotes

^1^ https://www.wjx.cn/
